# The ‘Wirral Wedge’: an aid to position arm safely in upper limb surgery

**DOI:** 10.1308/rcsann.2023.0103

**Published:** 2024-04-02

**Authors:** J Russell, J Holt, R Chandrasekar

**Affiliations:** ^1^ Countess of Chester Hospital NHS Foundation Trust, UK; ^2^ Wirral University Teaching Hospital NHS Foundation Trust, UK

## Background

Upper limb surgery such as arteriovenous (AV) fistula creation is increasingly being performed under regional anaesthetic techniques, with the patient awake.^1^ Patient comfort is maintained by positioning the operating table in the semi-upright position, usually at 30° inclination, and also a pillow under the knees. The arm-table is attached to the railings on the sides of the upper half of operating table. With this semi-upright position of the operating table, the surface of the arm-table is inclined downwards. When the limb-to-be-operated on is placed on this arm-table, the limb slides down. The surgical assistant is often required to restrain the arm in the externally rotated position.

## Innovation

**Figure 1:**
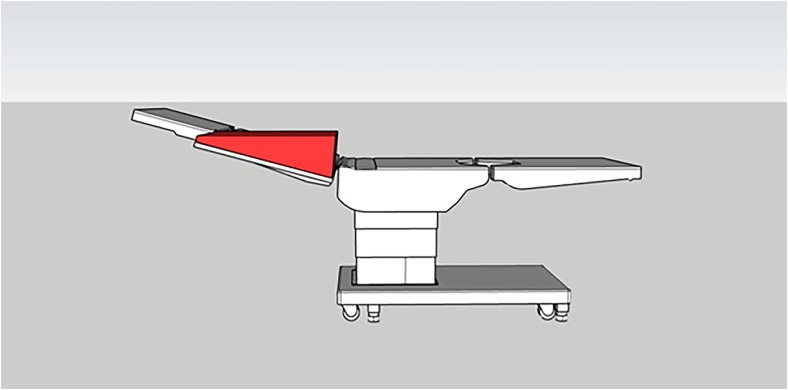
Reconstruction of an operating table with the device (highlighted in red) attached to the arm-table (lateral view)

**Figure 2:**
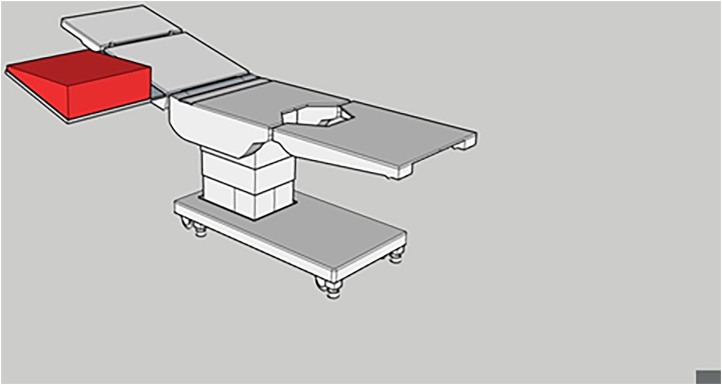
Reconstruction of an operating table with the device (highlighted in red) attached to the arm-table (oblique view)

We present a device (Figures 1 and 2) that can be used to maintain the arm in a horizontal position relative to the floor, without affecting the overall semi-upright position of the patient. It is a foam wedge that can be securely fastened to the arm-table and easily removed when not needed. The surface of the wedge enables the arm to be placed horizontally, in relation to the floor, thereby negating the risk of the arm sliding down. Use of the device means that the assistant is not required to restrain the arm during the operation and is therefore granted more manual freedom to assist with the procedure.

## Conclusion

Our experience of this device is that it is a simple, cheap and effective means of providing a comfortable and safe position for the arm during AV fistula creation.
